# Timing of preventive behavior in the case of a new and evolving health risk: the case of COVID-19 vaccination

**DOI:** 10.1186/s13561-024-00484-9

**Published:** 2024-02-27

**Authors:** Deeksha Gupta, Caroline Rudisill

**Affiliations:** Department of Health Promotion, Education, and Behavior, Arnold School of Public Health, University of South Carolina, Columbia, SC 29208 USA

**Keywords:** COVID-19, Preventive behavior, New risk, Vaccination timing

## Abstract

**Background:**

Time preferences for preventive behavior under novel risks and uncertain contexts may differ from timing preferences related to familiar risks. Therefore, it is crucial to examine drivers of preventative health behavior timing in light of new health risks. Using the case of COVID-19, we examine factors affecting vaccination timing plans when vaccines were widely available in the European Union (EU).

**Methods:**

We use data from the Flash Eurobarometer 494 survey (May 21–26, 2021), which collected information on EU residents’ attitudes towards COVID-19 vaccinations. We also use the ‘Our World in Data’ vaccination database for country-level COVID-19 vaccination rates. Probit regressions were conducted to determine how local vaccination rates, trust in information sources, social norms, vaccine safety beliefs, and risk understanding affected the probability of COVID-19 vaccination delay.

**Results:**

Of total participants (*n* = 26,106), 9,063 (34.7%) were vaccinated, 7,114 (27.3%) wanted to get vaccinated as soon as possible, 5,168 (19.8%) wanted to delay vaccination and 2,962 (11.4%) resisted vaccination. Participants were more likely to delay COVID-19 vaccination if they lived in a country with lower vaccination prevalence, trusted online social networks, family, friends, and colleagues for vaccination information, were eager to follow vaccination-related social norms, expressed vaccine safety concerns, and understood the risk of catching COVID-19 without a vaccine to be lower.

**Conclusions:**

Results from the study contribute to understanding important factors that predict timing of vaccination plans. These findings can also contribute to the wider knowledge base about timing of preventive behavior uptake in novel risk contexts.

**Supplementary Information:**

The online version contains supplementary material available at 10.1186/s13561-024-00484-9.

## Introduction

Understanding decision-making under risk and uncertainty constitutes a crucial component to predict and encourage health-related behaviors. Particularly in prevention, where costs associated with such behaviors appear earlier than benefits in the cost–benefit profile, timing is crucial as behavior and behavioral change may not appear until too late. COVID-19 vaccine is one such context. Vaccination (including boosters) needs to happen today to prevent higher likelihood of hospitalization and even death. It is, therefore, important to understand what factors affect the timing of COVID-19 vaccine uptake decisions. Those individuals who continue to resist or plan to delay vaccination are of key policy interest. The COVID-19 context is instructive because it constitutes a novel risk and understanding about time preferences for preventative behavior regarding novel risks is limited.

Subjective judgment about disease risk can impact decision-making for undertaking a related preventive action. Such risks are assessed based on various factors, including actual and perceived susceptibility to infection and underlying disease severity [1]. Previous studies have examined how trust in government, health professionals, friends, and family, social norms, and vaccine safety concerns affect preventive actions for diseases like influenza [2, 3]. However, in the case of what was first a novel health risk like the COVID-19 virus, information originally was lacking and then developed over time with lingering uncertainty regarding the disease threat. As a result, decision-making for preventive action related to a new health risk may differ from a known and familiar risk [4]. It is, therefore, important to understand the determinants of timing for preventive behavior uptake in the context of new and evolving health risks.

In 2020, the COVID-19 pandemic led to unprecedented efforts in vaccine development and testing across the globe. COVID-19 vaccines reduce the risk of hospitalization [5] and death due to severe infection [6]. However, more than a year since vaccines were made widely available, striking differences in COVID-19 vaccination rates as well as vaccine hesitancy continue to remain across the European Union (EU) [7, 8]. Therefore, overcoming vaccine hesitancy and delay is imperative to reduce the global spread of COVID-19 as well as hospitalizations and deaths.

To this end, we use the case of COVID-19 to understand factors associated with the timing of individuals’ preventive behaviors in the face of a novel risk. We examine delaying or not seeking COVID-19 vaccination when this vaccination is available. Specifically, we investigate how key factors that might be expected to impact COVID-vaccination influence the timing of this preventative behavior decision. We examine how trust in various sources of COVID-19 vaccine information, social norms associated with vaccination, COVID-19 infection risk understanding, beliefs about COVID-19 vaccine safety, and local vaccination rates affect the timing of preferences for COVID-19 vaccination.

## Background and conceptual framework

### State of the art on COVID-19 vaccination predictors

Since the rollout of COVID-19 vaccines, several studies have provided evidence for the role of trust, social norms, risk understanding and beliefs about vaccine safety in predicting vaccine uptake behavior. Perhaps one of the most crucial predictors of vaccine uptake behavior is trust in sources for vaccination information. The COVID-19 pandemic has been labeled as ‘pandemic of mistrust’ [9], fueled by conflicting public health recommendations, contrasting political and scientific opinions about the pandemic and misinformation regarding vaccine safety. Previous studies have highlighted how trust in government and other sources of information for COVID-19 predict vaccine uptake behavior. Brailovskaia and colleagues (2021) examined willingness to get vaccinated against COVID-19 in nine countries, five of which were in the EU (France, Germany, Poland, Spain, and Sweden). Individuals who obtained COVID-19-related information from news reports, print media, and official websites were more likely to get vaccinated against COVID-19, while the use of social media was associated with vaccine non-compliance [10]. The authors also found that individuals who perceived their country’s government as honest and credible were more likely to get vaccinated. Another study examining vaccine hesitancy found similar results for the EU when analyzing the survey data used in this article, Flash Eurobarometer 494 [11]. Trust in government, the EU, and health professionals predicted vaccine compliant behavior while trust in the internet, online social network, and other people predicted vaccine hesitancy with differences in findings across the European regions. However, trust in information from other people around was no longer significant upon controlling for beliefs about vaccines in general [11].

Concerns about COVID-19 vaccine safety and effectiveness also contribute to vaccination delay and hesitancy. A national survey conducted in the US found that individuals who believed COVID-19 vaccines were unsafe were less likely to get vaccinated, despite vaccine availability, and more likely to delay vaccination by four months at the least [12]. Similar results were found for studies conducted in the EU where concerns about potential side effects of COVID-19 vaccines [13] and general mistrust in vaccine safety and effectiveness contributed to COVID-19 vaccination hesitancy in two studies using the Flash Eurobarometer 494 [11, 14].

Finally, previous studies also demonstrate how social norms impact decision making for COVID-19 vaccinations. Wang and colleagues (2022) conducted a discrete choice experiment to determine factors affecting parental willingness to get their child vaccinated with available COVID-19 vaccines. Social acceptability of vaccines for children, indicated by vaccination coverage, had a positive effect on parental vaccine acceptability [15]. A study conducted among college students found similar results wherein perceived social norms for COVID-19 vaccinations was associated with vaccine hesitancy, after accounting for past infection from COVID-19, risk understanding, and pandemic related stress [16]. Studies conducted in EU countries provide similar support for social norms in predicting COVID-19 vaccination behaviors [8, 17].

### Time preferences for preventive behaviors and COVID-19

Since the availability of COVID-19 vaccines does not guarantee its uptake, it is crucial to understand the underlying causes of time preferences for COVID-19 related preventive behaviors and/or vaccine uptake- specifically related to delay and non-compliance. Previous research demonstrates the role of distrust in health authorities, safety concerns, risk perceptions and social norms in delaying or resisting preventive actions such as undergoing mammogram screening and getting vaccinated against H1N1 virus [18–21]. Similar evidence can be found for COVID-19-related preventive behaviors. Specifically, greater temporal discounting, i.e., tendency to place greater value in immediate rewards than future ones, and greater risk taking has been shown to increase the likelihood of resisting preventive actions such as mask wearing and social distancing [22]. Studies also support the role of trust in authorities, subjective norms, risk perception, perceived threat, and negative emotions such as worry in compliance with preventive behaviors (e.g. hand washing, covering mouth and nose while sneezing, avoiding close contact and social distancing) [23–25].

### Conceptual framework

**Fig. 1:**
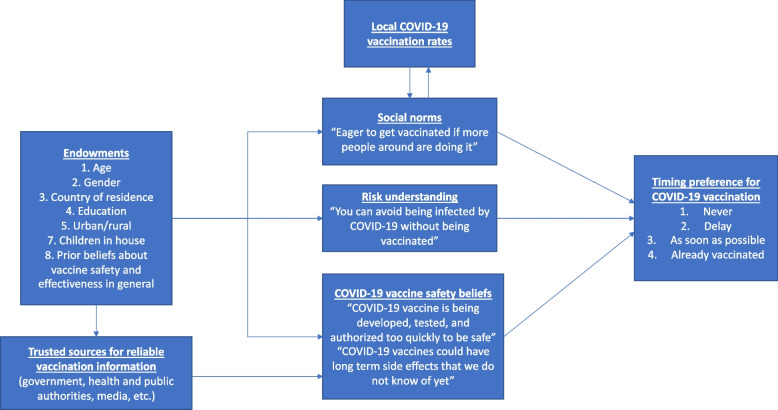
Conceptual framework for determinants of COVID-19 vaccination timing preference

Figure 1 depicts the conceptual framework used to guide our empirical specification for COVID-19 vaccination timing preference. Individuals have a certain set of endowments that might impact their COVID-19 vaccination timing preferences. These include age, gender, employment, having children at home and rural/urban residence [26, 27]. Prior beliefs about vaccine safety and effectiveness in general would also influence response to a new risk [11, 14] but these beliefs can also be specific to COVID-19 vaccines as conceptualized here. These endowments also shape an individual’s settings and social groups from whom they receive social cues regarding vaccination. Further, while social norms affect local vaccination prevalence, the latter, in turn, provides cues about societal acceptance of vaccines. Social norms impact vaccination uptake via the bandwagoning effect- as more people in an individual’s social group become vaccinated, signaling vaccination as an acceptable behavior, individuals tend to get vaccinated to fit in with their respective social group [28]. However, the salience of such social cues varies by the social group used as a reference (e.g., friends and family vs. coworkers, religious community) [29].

Endowments also determine an individual’s trust in different information sources for obtaining COVID-19 vaccination-related information and in turn, this information is used to update understanding of vaccine safety. Trust in COVID-19 vaccination information sources shape vaccine safety beliefs which can impact risk understanding about vaccines and COVID-19 vaccination timing.

## Methods

### Data

This study uses data from the Flash Eurobarometer Survey 494 [30], conducted among 26,106 residents of 27 EU countries aged 15 years and older, to collect information regarding attitudes towards COVID-19 vaccination. Flash Eurobarometer surveys are a part of the Eurobarometer survey series, conducted on behalf of the European Commission, to collect public opinions on a variety of topics of importance to public policy in the EU. The Flash Eurobarometers are small-scale surveys focused on special topics or target population groups and are conducted via telephone or online interviewing over a short period. The Flash Eurobarometer Survey 494 was conducted through computer-based web interviewing from May 21–26, 2021, i.e., about five months after the first COVID-19 vaccine was made available across the EU [31].

Quota sampling based on age, geographic region, and gender was used to recruit participants. About 1000 participants were recruited from each country, except Luxembourg, Cyprus, and Malta (~ 500 individuals participated in the survey in these countries) to obtain a total sample of 26,106 participants.

We also obtained local COVID-19 vaccination rates during the survey time period for each EU country from the Our World in Data dataset [32]. The vaccination database compiles official data from various sources such as national governments, health ministries, World Health Organization, research institutes and online databases. The COVID vaccination rates used in this analysis were based on individuals receiving at least one dose of a COVID-19 vaccine. Although most COVID-19 vaccines in the EU required two doses, we did not exclude individuals who received only the first dose. This is because vaccines were authorized only five months prior to the study period, and it is possible that many people did not receive the second dose in time. We used the earliest available vaccination rate for each country in the Eurobarometer sample from May 21–26, 2021, to match the date range of the Eurobarometer survey. This dataset was extracted by our team from Our World in Data on March 17, 2022.

### Methods

Studies eliciting time preferences for vaccine uptake use a number of methods, such as ad-hoc preferences [33] and choice-based experiments [34, 35]. However, the use of such elicitation methods to understand timing preferences is debated in research. For example, ad-hoc preferences may not predict actual preferences reliably because commodities associated (e.g., money) and discount rates differ from vaccinations [33]. Similarly, attributes assumed to contribute to preferences in choice-based experiments may differ from attributes used to make real-life decisions. Our study addresses this gap in time preference elicitation methods using a survey question that inquires of EU residents about their timing preference for COVID-19 vaccination.

The key outcome variable of interest in this study captures the timing of COVID-19 vaccination behaviors and plans; “When would you like to get vaccinated against COVID-19 (coronavirus)?”. Possible responses included “as soon as possible”, “sometime in 2021”, “later”, “never”, “I have already been vaccinated”, and “don’t know”. As our focus is vaccination timing, we combined responses such that individuals were said to (1) “delay” their vaccination if their response was either “sometime in 2021” or “later”, (2) be “vaccine compliant” if their response was either “as soon as possible” or “I have already been vaccinated”, (3) be “vaccine non-compliant” if their response was either “never” or “don’t know”. Thus, we examined six indicator outcome variables with the first level given the value of 1 and the latter 0: (1) delay vs. already vaccinated, (2) delay vs. as soon as possible, (3) delay vs. vaccine compliant, (4) never vs. delay, (5) don’t know vs. delay, (6) vaccine non-compliant vs. delay.

Independent variables of interest were obtained from both the Eurobarometer survey and the Our World in Data dataset. Local vaccination rates were obtained from the Our World in Data vaccination database and calculated from the number of vaccinated individuals and total population in a country from May 21–26, 2021. If the number of vaccinated individuals in a country varied between these days, we used figures for the earliest date during that period.

The remaining independent variables came from the Eurobarometer survey. Trust in information sources was determined from yes/no responses to the question, “Among the following sources, which ones would you trust more to give you reliable information on COVID-19 vaccines?”. Sources included the EU, national government, national health authorities, regional or local public health authorities, health professionals, doctors, nurses, and pharmacists, media (television, radio, newspapers), websites, online social networks, people around you (colleagues, friends, and family).

Social norms were determined from yes/no responses to the survey question, “You would be more eager to get vaccinated against COVID-19 if you see more people around you doing it”.

Participants also responded to COVID-19-specific vaccine safety statements, “COVID-19 vaccines are being developed, tested, and authorized too quickly to be safe” and “COVID-19 vaccines could have long term side effects that we do not know yet”. Possible responses included “totally agree”, “tend to agree”, “totally disagree”, “tend to disagree”, and “don’t know”.

Participant’s risk understanding was determined from the statement, “You can avoid being infected by COVID-19 without being vaccinated” wherein individuals could respond “totally agree”, “tend to agree”, “totally disagree”, “tend to disagree”, or “don’t know”. For analysis purposes, we combined the responses “totally agree” or “tend to agree” to “agree” and “totally disagree” or “tend to disagree” to “disagree” for the above-mentioned survey questions. Participant demographics included age, gender, age when full-time education was stopped, type of community of residence, and whether they had any children aged less than 15 years old in the household.

### Analysis

The conceptual framework presented above underpins the analytical strategy set forward. All variables in the conceptual framework are observable except prior beliefs about vaccine safety and effectiveness in general. Although prior beliefs are important predictors of vaccination timing preferences, we did not include these variables in our analysis due to multicollinearity issues. We used two-sample group proportions tests to determine differences in vaccination delay by local vaccination rates, trust in vaccine information sources, social norms, vaccine safety, and risk understanding.

We used binomial probit regressions to determine the probability of delaying or resisting COVID-19 vaccination:

$${Y}_{i}={\beta }_{1}VAXRAT{E}_{i}+{\beta }_{2}TRUS{T}_{i}+{\beta }_{3}SOCIA{L}_{i}+{\beta }_{4}SAFET{Y}_{i}+{\beta }_{5}RIS{K}_{i}+{\beta }_{6}DE{M}_{i}+{\epsilon }_{i}$$

We examined the six outcomes described earlier. Model specifications remained the same for each set of binary outcomes. Independent variables included local vaccination rate ($VAXRAT{E}_{i}$), a vector representing trust in public and health entities ($TRUS{T}_{i}$), social norms ($SOCIA{L}_{i}$), a vector representing participants’ responses to the two COVID-19 vaccination safety questions ($SAFET{Y}_{i}$), participants’ risk understanding associated with COVID-19 vaccination ($RISK_{i}$) and participant demographics ($DE{M}_{i}$). Standard errors are robust and clustered based on country of residence. Clustering by country accounts for heteroskedasticity in the error term by participants in individual countries having related responses to questions in ways not captured by this study’s explanatory and control variables and thereby overstating error terms. All models were tested for multicollinearity with the requirement of variance inflation factors (VIF) less than 10. Regression results are reported using average marginal effects (AME).

## Results

### Descriptive statistics

Descriptive statistics appear in Table 1. Participants (*n* = 26,106) were primarily aged between 25 to 39 years (6,803, 26.1%) or 50 to 64 (6,558, 25.1%), female (13,471, 51.6%), at least 20 years old (12,119, 46.4%) or 16–19 years old (7,962, 30.5%) when their full-time education stopped, lived in a town or city (19,589, 75.0%), and did not have a child in their household (17,046, 65.3%). On average, the local vaccination rate (in percentage) across all countries was 34.4 $\pm$ 8.62 with a range of 11–58%.

**Table 1:** Descriptive statistics for dependent and independent variables (*N* = 26,106)

VARIABLES	n	Mean	Std. Dev	Range^a^
***Dependent variable***				
When would you like to get vaccinated against COVID-19 (coronavirus)?				
As soon as possible	7,114	0.273	0.445	
Delay	5,168	0.198	0.398	
Never	2,962	0.113	0.317	
I have already been vaccinated	9,063	0.347	0.476	
Don’t know	1,460	0.056	0.230	
Prefer not to answer	339	0.013	0.113	
***Independent variables***				
Local vaccination rates (in percent)^b^	27	34.4	8.62	11.0 – 58.0
*Trust in public and health entities*				
Among all the following sources, which ones would you trust more to give you reliable information on COVID-19 vaccines?				
European Union	5,999	0.230	0.421	
National government	4,763	0.182	0.386	
National health authorities	11,442	0.438	0.496	
Local and regional public authorities	3,266	0.125	0.331	
Health professionals, doctors, nurses, and pharmacists	15,818	0.606	0.489	
Media (television, radio, newspapers)	2,815	0.108	0.310	
Websites	2,080	0.080	0.271	
Online social networks	1,483	0.057	0.231	
People around you (colleagues, friends, and family)	4,049	0.155	0.362	
*Social norm*				
You would be more eager to get vaccinated against COVID-19 if you see more people around you doing it				
Yes	3,729	0.143	0.350	
No	22,377	0.857	0.350	
*Vaccine safety*				
To what extent do you agree or disagree with each of the following statements?				
COVID-19 vaccines are being developed, tested, and authorized too quickly to be safe				
Totally agree/tend to agree	14,337	0.549	0.498	
Totally disagree/tend to disagree	9,744	0.373	0.484	
Don’t know	2,025	0.078	0.267	
COVID-19 vaccines may have long term side effects that we do not know yet				
Totally agree/tend to agree	16,817	0.644	0.479	
Totally disagree/tend to disagree	5,975	0.229	0.420	
Don’t know	3,314	0.127	0.333	
*Risk understanding*				
To what extent do you agree or disagree with each of the following statements?				
You can avoid being infected by COVID-19 without being vaccinated				
Totally agree/tend to agree	12,486	0.478	0.500	
Totally disagree/tend to disagree	11,181	0.428	0.495	
Don’t know	2,439	0.093	0.291	
*Demographics*				
Age (in years)				
15 to 24	3,597	0.138	0.345	
25 to 39	6,803	0.261	0.439	
40 to 49	4,641	0.178	0.382	
50 to 64	6,558	0.251	0.434	
65 and above	4,507	0.173	0.378	
Gender				
Male	12,549	0.481	0.500	
Female	13,471	0.516	0.500	
In another way	64	0.002	0.049	
Prefer not to answer	22	0.001	0.029	
Age when full time education was stopped				
Up to 15 years	809	0.031	0.173	
16 to 19 years	7,962	0.305	0.460	
20 years or older	12,119	0.464	0.499	
Still in full time education	2,952	0.113	0.317	
Never been in full time education	639	0.024	0.155	
Don’t know	1,184	0.045	0.208	
Refusal	441	0.017	0.129	
Type of community				
Rural area or village	6,517	0.250	0.433	
Town/city	19,589	0.750	0.433	
Children in house				
Yes	7,433	0.285	0.451	
No	17,046	0.653	0.476	
Don’t know	534	0.020	0.142	
Refusal	1,093	0.042	0.200	

^a^Range provided only for continuous variables

^b^Statistics for local vaccination rates are calculated at country-level and not for each individual

At the time of the survey, 9,063 (34.7%) participants were vaccinated, and 7,114 (27.3%) participants wanted to get vaccinated as soon as possible. 5,168 (19.8%) participants wanted to delay vaccination, and 2,962 (11.3%) participants did not want to get vaccinated.

The most trusted source of information on COVID-19 vaccines was health professionals, doctors, nurses, and pharmacists (15,818, 60.6%), followed by national health authorities (11,442, 43.8%) and then the EU (5,999, 23.0%). Most participants expressed that they would *not* be more eager to get vaccinated against COVID-19 if they saw others doing so (22,377, 85.7%). Related to COVID-19 vaccine safety beliefs, most participants had safety concerns due to rapid development, testing, and authorization of the vaccines (14,377, 54.9%) or possible long-term side effects from vaccination (16,817, 64.4%). Participants also were more likely to agree (12,486, 47.8%) that they could avoid COVID-19 infection without vaccination than disagree.

### Two-sample group proportions test

Table 2 depicts two-sample proportions test examining differences in vaccination delay by local vaccination rates, trust in public and health entities, and social norms. Compared to participants who were already vaccinated, the proportion of participants delaying vaccination was significantly greater in countries with local vaccination rates less than 30% (*p* = 0.000), among those who did not trust the EU (*p* = 0.000), national government (*p* = 0.000), national health authorities (*p* = 0.000), local and regional public authorities (*p* = 0.003), or health professionals, doctors, nurses, and pharmacists (*p *= 0.000), those who trusted websites (*p* = 0.000), online social networks (*p* = 0.000), or people around them (*p* = 0.000), and those who were eager to get vaccinated if more people around them were getting vaccinated (*p* = 0.000).

**Table 2:** Proportion of participants delaying COVID-19 vaccination by local vaccination rates and trust in information sources

`	Group 1		Group 2		z	*p*-value
	N	Mean (Std. Dev.)	N	Mean (Std. Dev.)		
*Local vaccination rates*						
	Vaccination rate < 30%		Vaccination rate > 30%			
Delay vs. already vaccinated	2337	0.495 (0.500)	11894	0.337 (0.473)	-14.5	0.000
Delay vs. as soon as possible	1747	0.662 (0.473)	10535	0.381 (0.486)	-22.0	0.000
Delay vs. never	1917	0.603 (0.489)	6213	0.646 (0.478)	3.40	0.001
*Trust in public, health, and social entities for giving reliable COVID-19 vaccine information*						
	Trust in European Union		No trust in European Union			
Delay vs. already vaccinated	3480	0.318 (0.466)	10751	0.378 (0.485)	6.44	0.000
Delay vs. as soon as possible	3208	0.344 (0.475)	9074	0.448 (0.497)	10.2	0.000
Delay vs. never	1301	0.849 (0.358)	6829	0.595 (0.491)	-17.5	0.000
	Trust in national government		No trust in national government			
Delay vs. already vaccinated	2643	0.296 (0.457)	11588	0.378 (0.485)	7.93	0.000
Delay vs. as soon as possible	2606	0.300 (0.459)	9676	0.453 (0.498)	14.0	0.000
Delay vs. never	925	0.846 (0.361)	7205	0.609 (0.488)	-14.2	0.000
	Trust in national health authorities		No trust in national health authorities			
Delay vs. already vaccinated	6824	0.280 (0.449)	7407	0.439 (0.496)	19.7	0.000
Delay vs. as soon as possible	5779	0.331 (0.471)	6503	0.501 (0.500)	19.0	0.000
Delay vs. never	2242	0.853 (0.354)	5888	0.553 (0.497)	-25.2	0.000
	Trust in local and regional public authorities		No trust in local and regional public authorities			
Delay vs. already vaccinated	1769	0.332 (0.471)	12,462	0.368 (0.482)	2.93	0.003
Delay vs. as soon as possible	1879	0.312 (0.464)	10,403	0.440 (0.496)	10.3	0.000
Delay vs. never	696	0.843 (0.364)	7434	0.616 (0.486)	-11.9	0.000
	Trust in health professionals, doctors, nurses, and pharmacists		No trust in health professionals, doctors, nurses, and pharmacists			
Delay vs. already vaccinated	9407	0.313 (0.464)	4824	0.461 (0.499)	17.4	0.000
Delay vs. as soon as possible	7645	0.385 (0.487)	4637	0.480 (0.500)	10.3	0.000
Delay vs. never	3850	0.765 (0.424)	4280	0.520 (0.500)	-22.9	0.000
	Trust in media		No trust in media			
Delay vs. already vaccinated	1517	0.384 (0.487)	12,714	0.361 (0.480)	-1.81	0.070
Delay vs. as soon as possible	1583	0.368 (0.482)	10,699	0.429 (0.495)	4.53	0.000
Delay vs. never	752	0.775 (0.418)	7378	0.621 (0.485)	-8.35	0.000
	Trust in websites		No trust in websites			
Delay vs. already vaccinated	1014	0.475 (0.500)	13,217	0.354 (0.478)	-7.71	0.000
Delay vs. as soon as possible	1074	0.449 (0.498)	11,208	0.418 (0.493)	-1.95	0.052
Delay vs. never	802	0.601 (0.490)	7328	0.639 (0.480)	2.15	0.032
	Trust in online social networks		No trust in online social networks			
Delay vs. already vaccinated	701	0.572 (0.495)	13,530	0.352 (0.478)	-11.8	0.000
Delay vs. as soon as possible	793	0.506 (0.500)	11,489	0.415 (0.493)	-5.01	0.000
Delay vs. never	679	0.591 (0.492)	7451	0.640 (0.480)	2.55	0.011
	Trust in people around you		No trust in people around you			
Delay vs. already vaccinated	2116	0.518 (0.500)	12,115	0.336 (0.472)	-16.0	0.000
Delay vs. as soon as possible	2186	0.501 (0.500)	10,096	0.403 (0.491)	-8.42	0.000
Delay vs. never	1599	0.685 (0.464)	6531	0.623 (0.485)	-4.61	0.000
*Social norms- you would be more eager to get vaccinated if you see more people around you doing it*						
	Yes		No			
Delay vs. already vaccinated	2171	0.433 (0.496)	12,060	0.351 (0.477)	-7.30	0.000
Delay vs. as soon as possible	2251	0.417 (0.493)	10,031	0.422 (0.494)	0.386	0.699
Delay vs. never	1027	0.914 (0.280)	7103	0.595 (0.491)	-19.9	0.000

Results were similar when comparing the proportion of participants delaying vaccination and those who wanted to get vaccinated as soon as possible, except for social norms and trust in websites not being important and trust in media being important. The proportion of participants delaying vaccination was greater among those who did not trust media (*p* = 0.000) for receiving COVID-19 vaccine information. However, there were no significant differences in the proportion of participants delaying vaccination between those who did and did not trust websites for obtaining COVID-19 vaccination information (z = -1.95, *p* = 0.052) and between those who were eager to get vaccinated and those who were not, if others were getting vaccinated (z = 0.386, *p* = 0.699).

However, compared to participants who never wanted to get vaccinated, the proportion of participants who wanted to delay vaccination was smaller in countries with local vaccination rate less than 30% (*p* = 0.001), among those who did not trust the EU (*p* = 0.000), national government (*p* = 0.000), health authorities (*p* = 0.000), local and regional public authorities (*p* = 0.000), health professionals, doctors, nurses, and pharmacists (*p* = 0.000), media (*p* = 0.000), people around them (*p* = 0.000), and those who trusted websites (*p* = 0.032), and online social networks (*p* = 0.011), and those who were not eager to get vaccinated if more people around them were doing it (*p* = 0.000).

Table 3 compares differences in proportion of participants delaying vaccination by vaccine safety beliefs and risk understanding. Compared to participants who were already vaccinated or who wanted to get vaccinated as soon as possible, the proportion of participants delaying vaccination was greater among those who agreed that vaccines were being developed, tested and authorized too quickly to be safe (*p* = 0.000), who agreed that vaccines may have long term side effects (*p* = 0.000), and those who believed that they can avoid being infected without COVID-19 vaccination (*p* = 0.000). However, when comparing participants who did not want to get vaccinated, the proportion of those who wanted to delay vaccination was lower among those who agreed that vaccines were being developed, tested and authorized too quickly to be safe (*p* = 0.000), who agreed that vaccines may have long term side effects (*p* = 0.000), and those who believed that they can avoid being infected without COVID-19 vaccination (*p* = 0.000).

**Table 3:** Proportion of participants delaying COVID-19 vaccination by vaccine safety and risk understanding

Variables	Group 1		Group 2		z	*p*-value
	N	Mean (Std. Dev.)	N	Mean (Std. Dev.)		
*Vaccine safety- COVID-19 vaccines are being developed, tested, and authorized too quickly to be safe*						
	Agree/tend to agree		Disagree/tend to disagree			
Delay vs. already vaccinated	7286	0.507 (0.500)	5762	0.203 (0.403)	-35.6	0.000
Delay vs. as soon as possible	6948	0.532 (0.499)	4463	0.263 (0.440)	-28.4	0.000
Delay vs. never	6175	0.598 (0.490)	1573	0.745 (0.436)	10.8	0.000
*Vaccine safety- COVID-19 vaccines may have long term side effects that we do not know of yet*						
	Agree/tend to agree		Disagree/tend to disagree			
Delay vs. already vaccinated	8621	0.462 (0.499)	3571	0.222 (0.416)	-24.8	0.000
Delay vs. as soon as possible	8096	0.492 (0.500)	2814	0.281 (0.450)	-19.4	0.000
Delay vs. never	6636	0.601 (0.490)	1016	0.780 (0.415)	11.0	0.000
*Risk understanding- you can avoid being infected by COVID-19 without getting vaccinated*						
	Agree/tend to agree		Disagree/tend to disagree			
Delay vs. already vaccinated	5918	0.535 (0.499)	6945	0.219 (0.414)	-37.1	0.000
Delay vs. as soon as possible	6204	0.510 (0.500)	5003	0.304 (0.460)	-22.0	0.000
Delay vs. never	5519	0.573 (0.495)	1960	0.777 (0.417)	16.0	0.000

### Multivariate analysis

Table 4 presents the results from probit regressions and the average marginal effects of each independent variable on the outcomes. Marginal effects describe how a unit change in an independent variable is associated with change in the probability of early timing preferences for COVID-19 vaccination to delaying or resisting vaccination. Models 1–3 provide the results for COVID-19 vaccination delay versus already vaccinated (1), getting vaccinated as soon as possible (2), and vaccination compliance (3). Similarly, Models 4–6 describe the results for those who resist vaccination (4), who don’t know when they would get vaccinated (5), or are vaccine non-compliant (6), respectively, vs. those who want to delay COVID-19 vaccination. Results from Table 4 are explained below:

**Table 4:** Probit regression with average marginal effects (AME) for COVID-19 vaccination timing preference

	(1)	(2)	(3)	(4)	(5)	(6)
VARIABLES	Delay vs. Already	Delay vs. As soon as possible	Delay vs. Already + As soon as possible	Never vs. Delay	Don't know vs. Delay	Never + Don't know vs. Delay
	AME (Std. Err.)	AME (Std. Err.)	AME (Std. Err.)	AME (Std. Err.)	AME (Std. Err.)	AME (Std. Err.)
*Local vaccination rates (ref.* = *less than 30%)*						
30 to 40%	-0.058	-0.222**	-0.111**	-0.037	0.005	-0.026
	(0.055)	(0.041)	(0.040)	(0.028)	(0.022)	(0.027)
40% or more	-0.187	-0.101	-0.142*	-0.029	0.030	-0.004
	(0.096)	(0.054)	(0.063)	(0.062)	(0.030)	(0.054)
*Trust in sources for reliable COVID-19 vaccines information*						
Among all the following sources, which ones would you trust more to give you reliable information on COVID-19 vaccines?						
European Union	-0.038**	-0.039**	-0.034**	-0.145**	-0.060**	-0.128**
	(0.012)	(0.011)	(0.007)	(0.014)	(0.014)	(0.016)
National government	-0.012	-0.059**	-0.030**	-0.084**	-0.033	-0.063**
	(0.013)	(0.017)	(0.011)	(0.023)	(0.018)	(0.021)
National health authorities	-0.037**	-0.062**	-0.039**	-0.177**	-0.044**	-0.139**
	(0.010)	(0.011)	(0.007)	(0.011)	(0.011)	(0.012)
Local and regional public authorities	0.004	-0.039**	-0.014	-0.074**	-0.040	-0.066**
	(0.017)	(0.014)	(0.011)	(0.029)	(0.023)	(0.025)
Health professionals, doctors, nurses, and pharmacists	-0.045**	-0.030*	-0.030**	-0.160**	-0.048**	-0.137**
	(0.007)	(0.012)	(0.007)	(0.012)	(0.011)	(0.012)
Media (television, radio, newspapers)	0.017	-0.029*	-0.006	-0.048**	-0.020	-0.043*
	(0.011)	(0.012)	(0.007)	(0.017)	(0.016)	(0.017)
Websites	0.009	0.004	0.004	0.034	0.014	0.040*
	(0.018)	(0.018)	(0.012)	(0.020)	(0.014)	(0.016)
Online social networks	0.042**	0.017	0.023*	0.056*	-0.029	0.035
	(0.014)	(0.017)	(0.011)	(0.022)	(0.017)	(0.021)
People around you (colleagues, friends, and family)	0.043**	0.034*	0.035**	-0.048**	-0.001	-0.033*
	(0.013)	(0.014)	(0.008)	(0.015)	(0.014)	(0.014)
*Social norm*						
You would be more eager to get vaccinated against COVID-19 if you see more people around you doing it. (ref. = no)						
Yes	0.037**	0.002	0.016	-0.256**	-0.083**	-0.208**
	(0.013)	(0.014)	(0.009)	(0.025)	(0.017)	(0.019)
*Vaccine safety*						
To what extent do you agree or disagree with each of the following statements?						
COVID-19 vaccines are being developed, tested, and authorized too quickly to be safe. (ref. = totally disagree/tend to disagree)						
Totally agree/tend to agree	0.143**	0.151**	0.119**	0.032	0.013	0.031*
	(0.009)	(0.011)	(0.008)	(0.018)	(0.013)	(0.015)
Don’t know	0.040**	0.053**	0.036**	-0.042	0.074**	0.034
	(0.013)	(0.017)	(0.011)	(0.025)	(0.029)	(0.026)
COVID-19 vaccines could have long term side effects that we do not know yet. (ref. = totally disagree/tend to disagree)						
Totally agree/tend to agree	0.090**	0.097**	0.076**	0.083**	0.065**	0.100**
	(0.011)	(0.010)	(0.008)	(0.022)	(0.017)	(0.021)
Don’t know	-0.024	-0.016	-0.018	-0.044	0.086**	0.049
	(0.012)	(0.015)	(0.010)	(0.025)	(0.028)	(0.029)
*Risk understanding*						
To what extent do you agree or disagree with each of the following statements?						
You can avoid being infected by COVID-19 without being vaccinated. (ref. = totally disagree/tend to disagree)						
Totally agree/tend to agree	0.127**	0.111**	0.098**	0.128**	0.068**	0.127**
	(0.009)	(0.014)	(0.010)	(0.013)	(0.011)	(0.011)
Don’t know	0.093**	0.103**	0.079**	-0.003	0.140**	0.092**
	(0.013)	(0.012)	(0.008)	(0.025)	(0.016)	(0.020)
*Demographics*						
Age (ref. = 15–24 years)						
25–39 years	-0.115**	-0.037*	-0.051**	-0.002	-0.011	-0.003
	(0.018)	(0.017)	(0.012)	(0.020)	(0.015)	(0.020)
40–49 years	-0.223**	-0.097**	-0.117**	-0.021	-0.020	-0.019
	(0.019)	(0.020)	(0.014)	(0.019)	(0.022)	(0.022)
50–64 years	-0.328**	-0.115**	-0.177**	0.004	-0.025	-0.005
	(0.024)	(0.024)	(0.016)	(0.027)	(0.019)	(0.025)
65 years and above	-0.447**	-0.120**	-0.258**	-0.004	-0.039	-0.018
	(0.031)	(0.031)	(0.021)	(0.029)	(0.025)	(0.027)
Gender (ref. = female)						
Male	0.014	-0.019	-0.001	-0.017	-0.046**	-0.033**
	(0.008)	(0.012)	(0.007)	(0.010)	(0.011)	(0.011)
In another way	0.091	0.068	0.071	-0.049	-0.060	-0.072
	(0.100)	(0.073)	(0.061)	(0.100)	(0.101)	(0.113)
Prefer not to answer	-0.024	-0.092	-0.059	-0.007	0.189	0.134
	(0.163)	(0.123)	(0.091)	(0.164)	(0.100)	(0.105)
Age when full time education was stopped (ref. = 20 years or older)						
Up to 15 years	0.001	0.016	0.010	0.056	0.064	0.072
	(0.023)	(0.027)	(0.019)	(0.042)	(0.036)	(0.040)
16–19 years	0.013	0.022	0.014	0.037**	0.039**	0.045**
	(0.011)	(0.013)	(0.008)	(0.012)	(0.012)	(0.013)
Still in full time education	0.009	-0.029	-0.013	0.008	0.039*	0.035*
	(0.021)	(0.019)	(0.014)	(0.020)	(0.019)	(0.016)
Never been in full time education	0.075**	0.077**	0.064**	-0.003	-0.027	-0.016
	(0.026)	(0.028)	(0.020)	(0.033)	(0.041)	(0.031)
Don’t know	0.009	0.004	0.006	0.070**	0.124**	0.115**
	(0.019)	(0.024)	(0.015)	(0.020)	(0.027)	(0.027)
Refusal	0.008	0.000	0.002	0.120**	0.077*	0.120**
	(0.031)	(0.057)	(0.032)	(0.030)	(0.035)	(0.029)
Type of community (ref. = town/city)						
Rural	-0.011	0.001	-0.005	0.053**	0.010	0.040**
	(0.011)	(0.015)	(0.009)	(0.013)	(0.013)	(0.014)
Children in house (ref. = no)						
Yes	0.033**	0.005	0.015*	-0.005	-0.009	-0.009
	(0.011)	(0.009)	(0.007)	(0.012)	(0.011)	(0.012)
Don’t know	0.149**	0.067*	0.077**	0.007	-0.027	-0.011
	(0.034)	(0.034)	(0.026)	(0.036)	(0.023)	(0.033)
Refusal	0.043**	0.036	0.032*	0.034	0.033	0.041
	(0.016)	(0.025)	(0.015)	(0.024)	(0.029)	(0.023)
Observations	14,231	12,282	21,345	8,130	6,628	9,590
Pseudo R^2^	0.285	0.124	0.168	0.181	0.061	0.119
Log pseudolikelihood	-6663.08	-7326.20	-9828.79	-4369.42	-3282.06	-5829.99

Estimates are average marginal effects (AME). Standard errors in parentheses

^**^p *<* 0.01

^*^p < 0.05

#### Effect of local vaccination rates

Results in Table 4 indicate that greater local vaccination rates decreased the probability of delaying vaccination. Compared to countries with vaccination rates less than 30%, individuals residing in countries with vaccination rates between 30 to 40% were less likely to delay vaccination. The decrease in probability of vaccination delay with greater local vaccination rates was significant versus the timing preference of ‘as soon as possible’ (AME = -0.222, *p* = 0.000) and versus vaccination compliance (AME = -0.111, *p* = 0.005). Similarly, individuals residing in countries with local vaccination rates greater than 40% were less likely to delay vaccination versus be vaccine compliant (AME = -0.142, *p* = 0.023) (Model 5).

#### Effect of trust in sources for reliable COVID-19 vaccines information

Across all models, individuals who trusted the EU, national government, national health authorities, local and regional public authorities, health professionals, doctors, nurses, pharmacists, and media had a lower probability of delaying or resisting vaccination. Individuals who trusted websites for obtaining COVID-19 vaccination information were 3.96% more likely to be vaccine non-compliant (i.e., refuse to get vaccinated or don’t know when they would get vaccinated) than delay vaccination. Similarly, those who trusted online social networks and people around them were more likely to delay vaccination. Individuals who trusted people around them were 4.84% less likely to never get vaccinated versus delay and 3.32% less likely to not comply with vaccination (never + don't know) versus delay.

#### Effect of social norms

Individuals who were eager to get vaccinated if they saw more people around them getting vaccinated were 3.74% more likely to delay vaccination than have already received vaccination. Further, those who were eager to get vaccinated if people around them were getting vaccinated were more likely to delay vaccination than resist or not comply with it (columns 4 to 6). Further illustration of the impact of social norms on COVID-19 vaccination timing appears in Figs. 1–4 in Online Resource 1. These figures show how vaccination timing preferences are related to mean social norms responses at the country level and vaccination rates by country.

#### Effect of beliefs about vaccine safety

Concerns about vaccine safety played a significant role in determining the timing preference for COVID-19 vaccination. Individuals who agreed that the vaccines were being developed, tested, and authorized too quickly to be safe were more likely to delay vaccination than get vaccinated sooner. Further, those who agreed that vaccines were being developed, tested, and authorized too quickly were 3.09% more likely to never get vaccinated than delay vaccination or not know when they would get vaccinated. Individuals who agreed that vaccines may have long term side effects were more likely to delay vaccination than get vaccinated sooner and more likely to never get vaccinated than delay.

#### Effect of risk understanding

Compared to those who disagreed, individuals who agreed that they can avoid being infected from COVID-19 without being vaccinated had greater probability of delaying vaccination (vs. getting vaccinated sooner) or never getting vaccinated (vs. delaying vaccination).

#### Demographics

Age, gender, education, community type, and presence of children in house had significant association with probability of vaccination delay. Age was associated with lower probability of delaying vaccination. Compared to those aged between 15–24 years, participants aged 25–39 years were 3.7% less likely to delay vaccination than get vaccinated as soon as possible. The probability of delay in vaccination declined further with age and was robust across all models. Further, compared to female participants, male participants were 4.62% less likely to not know when they would get vaccinated and 3.34% less likely to be vaccine non-compliant. Regarding education level, compared to individuals who were 20 years old, those who were 16–19 years old when their full-time education stopped were 3.69% more likely to never get vaccinated, 3.87% more likely to not know when they planned to get vaccinated, and 4.53% more likely to be vaccine non-compliant. Similarly, those who never had full-time education were more likely to delay vaccination. Further, participants who lived in rural communities than towns/cities were 5.30% more likely to never get vaccinated and 4.00% more likely to be vaccine non-compliant. Finally, presence of children in the house increased the probability of delaying vaccination (vs. being already vaccinated) by 3.34%.

#### Sensitivity analyses

We also conducted sensitivity analyses to determine the probability of getting vaccinated as soon as possible (vs. already vaccinated) and the probability of getting vaccinated later (vs. in 2021) (Online Resource 2). Results indicated that individuals residing in countries with vaccination rates between 30 to 40% were more likely to get vaccinated as soon as possible (vs. already vaccinated) than those in countries with lower vaccination rates. Further, those who trusted national government had greater likelihood of getting vaccinated soon (AME = 0.036, *p* = 0.006) but less likely to delay it beyond 2021 (AME = -0.069, *p* = 0.003). The finding regarding the desire to get vaccinated as soon as possible contrasts with our earlier findings indicating lower vaccination rates and distrust in national government to be associated with delay in vaccination timing. A possible explanation for these results is that greater local vaccination rates and trust in government may increase individual’s willingness to get vaccinated but there is a delay in 'immediate action’ (i.e., actual vaccination uptake) due to factors such as vaccine availability and timing of COVID-19 infections.

Further, we also found that individuals who trusted health professionals were 2.7% less likely to get vaccinated soon (vs. already vaccinated) and 4.5% less likely to get vaccinated later (vs. in 2021). Similarly, those who trusted public authorities and media were 5.1% and 3.3%, respectively, more likely to get vaccinated as soon as possible. Getting vaccinated later than 2021 was also associated with lower probability of trust in national health authorities (AME = -0.107, *p* = 0.000) but greater probability of trust in colleagues, friends, and family (AME = 0.030, *p* = 0.023). Further, individuals who saw other people around them getting vaccinated had greater likelihood of getting vaccinated soon (AME = 0.044, *p* = 0.002) and were less likely to delay it later than 2021 (AME = -0.119, *p* = 0.000). Individuals who believed that vaccines were not safe and had long term side effects were more likely to get vaccinated soon (vs. already vaccinated) or later (vs. in 2021). These individuals also believed that they could avoid getting infected without being vaccinated. The above findings are consistent with our previous results.

## Discussion

This study aims to understand the determinants of COVID-19 vaccination timing using a large EU-wide survey conducted when COVID-19 vaccines were widely available. We focus our analysis on those who delay vaccination versus vaccinating immediately or as soon as possible upon vaccine availability. Our study is distinguished from those examining COVID-19 vaccine hesitancy [8, 10–17] and analyzing factors associated with COVID-19 vaccination decisions because of our focus on COVID-19 vaccination timing and specifically delay in COVID-19 vaccination. The survey data used allows for assessment of different levels or severities of delay (later vs sometime in 2021 and as soon as possible vs already vaccinated). The COVID-19 vaccination decision provides an exemplar context where individuals are making a preventive decision regarding a novel health risk and thus provides insight into the drivers of time preferences in such a context. We test the hypothesis that trust in information sources, local vaccination rates, social norms, risk understanding and beliefs about vaccine safety may all play a role in timing preferences for COVID-19 vaccination. We take advantage of the wording of the COVID-19 vaccination question in the survey to delineate the people who delay vaccination versus those who have no plans to vaccinate or those who already have, which are the traditional groups of analysis. This population has not made up their mind enough to say ‘no’ but also not felt compelled to say ‘yes.’ This illustration of time preferences presents a unique setting to understand preventive decision-making in the face of a novel risk where individuals’ stock of information and experiences about the virus has increased over time.

The negative relationship between likelihood of COVID-19 vaccination delay, local vaccination rates and social norms demonstrates the role social environment plays in impacting vaccination decisions. While the sample population overwhelmingly stated that social norms (whether other people’s decision influenced their vaccination decision) would not impact their COVID-19 vaccination decision, we find evidence that it does indeed predict vaccination timing preferences. Specifically, we observe the association between social norms and resisting or not complying with COVID-19 vaccinations. Similar to our study, Algan et al. (2021) also found low compliance for COVID-19 restrictions in countries like Sweden where social trust was high, possibly resulting from the belief that others would comply with restrictions [36]. Sweden’s less restrictive lockdown policy during the initial pandemic wave may have shaped social norms among residents, resulting in fewer individuals working from home, avoiding contact with others and avoiding public transport compared to residents in analogous countries [37]. Although we expect greater vaccination compliance signaled by social norms, studies have shown that free riding behavior can reduce such bandwagoning effects from social norms [28]. Preference to free ride by delaying own vaccination and attaining benefits from others getting vaccinated (e.g., through herd immunity) has been observed in previous studies [38]. Moreover, with a new health risk, individuals also have limited information about the vaccine side-effects and therefore, prefer to “wait and see” and then reassess their own cost and benefits associated with vaccination [35, 39]. Given rates of concerns about vaccine safety in our sample, it is a possibility that such a process is occurring in this sample.

We also found that individuals who believe that vaccines were not safe were more likely to delay vaccination or resist vaccination. Existing studies have provided similar evidence about the association between vaccine safety perceptions and vaccination uptake for diseases such as influenza [40] and the H1N1 flu [41]. Moreover, concerns about vaccine safety and effectiveness in general have been found to be associated with COVID-19 vaccine hesitancy [11] and COVID-19 vaccination [14] in the sample studied here. Perceptions about vaccine safety impact risk appraisal such that in case of safety concerns, risks associated with vaccination outweighs its benefits. Previous studies have shown that for new health risks, perceived risk is greater than known risk and individuals are more likely to adopt avoidance behavior [42]. In case of COVID-19, the novelty of the disease and the unprecedented rate at which the vaccines were developed may have contributed to the overestimation of perceived risk from vaccination and therefore, greater likelihood of delaying vaccination.

Our study’s findings also suggest greater likelihood of delaying vaccination among those who believe they can avoid COVID-19 infection without vaccinations. Perceived susceptibility to a disease, defined as beliefs about probability of getting infected from a disease, has been theoretically linked to vaccine uptake behavior. Studies have shown that with lower perceived susceptibility to disease, individuals perceive the threat of disease and/or its symptoms to be low and therefore, avoid getting vaccinated. However, with new health risks, such as COVID-19, there is mixed evidence for the relationship between perceived susceptibility to the disease and vaccine uptake [43]. To this end, our study adds to the growing evidence regarding perceived susceptibility to a disease and vaccine uptake behavior.

Finally, we also find that the likelihood of vaccination delay varied based on sources trusted for information about COVID-19 vaccines. Specifically, individuals were more likely to be vaccine compliant (i.e., not delay vaccination) if they trusted health and public institutions and traditional media sources and less likely to be vaccine compliant (i.e., delay vaccination) if they trusted online social networks, colleagues, friends, and family. These results are largely consistent with existing analysis of this dataset focusing on vaccine hesitancy with some notable exceptions [11]. We see no relationship between trust in websites and vaccine delay as was seen with vaccine hesitancy. In addition, we observe a robust positive association between trusting colleagues, friends and family and vaccine delay, which was not observed for vaccine hesitancy. In case of a novel risk like COVID-19, where knowledge pertaining to efficacy of preventive action was continuously being produced and happening in real time, individuals seek information from various sources. However, the salience of such information in promoting vaccination varies between different sources and prevalence of different information types. For example, on the one hand, individuals often seek vaccine safety and effectiveness information from credible sources such as health and public authorities and media where information is regulated and presented strategically to promote preventive behaviors. On the other hand, individuals are more likely to uptake narrative health information than scientific information via online social networks [44, 45] and others including their colleagues, friends, and family to determine perceived risk. Further, information shared via such sources is often difficult to monitor and interpret for credibility leading to a greater prevalence of misinformation and information overload which affects perceived risk [46] and preventive behavior adoption. Nevertheless, our findings suggest that social media can be augmented to disseminate information in a narrative format by a credible and/or relatable individual, potentially improving vaccination uptake. Notably, such health communication strategies have previously been used to promote positive discourse regarding human papillomavirus vaccination [47]. Tailoring public health messages to specific demographics and communities [48] and utilizing media messaging tailored to specific population groups has also shown potential to improve influenza vaccination uptake [49].

Our study has some limitations. First, we are not able to make causal inferences between timing of vaccine uptake behavior and the explanatory factors examined here. The data used in this study is cross-sectional in nature and thus does not capture how people acquire information over time and update risk understanding and other features found important to decision-making at this moment in time when surveyed. Future studies should examine temporal effects of these factors on vaccination behavior, especially since vaccination preferences are known to change over time [50]. Second, we found that except for model 5, all specifications had omitted variable bias. This is likely due to omission of unobserved factors that influence timing of vaccination directly or via covariates in our specification (e.g., personal experience with COVID-19, employment type). Country-related factors such as vaccine roll-out strategies and information campaigns would be expected to be captured via clustering observations by country of residence. We anticipated that important predictors of vaccination behavior timing such as opinions about vaccine safety and effectiveness in general would be a source of omitted variable bias but correlation values with vaccine timing were very low in preliminary descriptive analysis (correlation range: 0.064 $\ge$ r $\ge$ 0.174, *p* < 0.01, see Online Resource 3 for further details). These variables were excluded from our models due to multicollinearity issues. Third, we used binomial regression models because we were unable to compare all vaccination timing preferences simultaneously. We found that multinomial vaccination timing preferences model violated the independence of irrelevant alternatives assumption, i.e., individuals perceived vaccination timing choices to be very similar. Although using binomial models reduced our sample size for each individual model, the sample size of our study was large, so the models still have large enough sample for obtaining conclusive results across covariates. Fourth, since vaccination timing preferences were self-reported, participant responses may have a social-desirability bias. Relatedly, actual vaccination outcome may differ from vaccination timing preferences reported in the study.

## Conclusion

Our study offers some important findings and policy implications for communications and expectations about preventive behavior regarding health risks. Our findings suggests that in the case of new health risks, trust in health and public authorities, media, and online platforms (but not websites), social norms, perceptions about vaccine safety, and risk understanding play crucial roles in the likelihood of delaying preventative behavior. Therefore, social norms persist even if people think that social norms do not matter. In getting people to move from a state of delay to action, these findings suggest that with an increase in the use of technology during and now as the pandemic wanes, policymakers can utilize online social platforms to share scientific and narrative information on COVID-19 vaccination. Innovations in strategies are needed to overcome these timing preferences and mitigate adverse health impacts related to COVID-19 and any future pandemics.

### Supplementary Information

**Additional file 1. Online Resource 1.** Relationship between vaccination timing preference, social norms and vaccination rates by country.

**Additional file 2. Online resource 2.** Sensitivity analysis for COVID-19 vaccination timing categories.

**Additional file 3. Online resource 3*.*** Correlation between vaccination attitudes in general and the timing of COVID-19 vaccinations*.*

## Data Availability

The data used in this study are available at the GESIS Data Archive for the Social Sciences (https://doi.org/10.4232/1.13786) and Our World in Data website (https://ourworldindata.org/covid-vaccinations).

## Abbreviations

EU: European Union
VIF: Variance inflation factor
AME: Average marginal effect
