# Supplementary material for: Timing of preventive behavior in the case of a new and evolving health risk: the case of COVID-19 vaccination
Source: Health Econ Rev. 2024 Feb 27;14:16. doi: 10.1186/s13561-024-00484-9 (PMC11344437; doi:10.1186/s13561-024-00484-9)
Supplement: Supplementary file 1 — Additional file 1. Online Resource 1. Relationship between vaccination timing preference, social norms and vaccination rates by country. [file 13561_2024_484_MOESM1_ESM.docx]

**Online Resource 1: Relationship between vaccination timing preference, social norms and vaccination rates by country**

The below figures and table show how vaccination timing preferences are related to mean social norms responses at the country level and vaccination rates by country. We examined the relationship between the proportion of sample delaying or resisting COVID-19 vaccination and local vaccination rates by social norms. Figures 1, 2, and 4 depict the relationship between local COVID-19 vaccination rates and proportion of sample delaying COVID-19 vaccination (vs. already vaccinated), never getting vaccinated (vs. delaying vaccination), and not complying with vaccination-i.e., never or not knowing when to get vaccinated (vs. delaying vaccination), respectively. Each of these figures demonstrate that as local vaccination prevalence increases, proportion of sample delaying or resisting vaccination decreases. In figure 1, for participants who are not eager to follow COVID-19 related social norms, the proportion of sample delaying vaccination decreases at a greater rate (slope coefficient = -1.18) than those who are eager to follow social norms (slope coefficient = -1.16). In figure 2, for participants who are eager to follow social norms, the proportion of sample never getting vaccinated decreases at a greater rate (slope coefficient = -0.242) than those who are not eager to follow social norms (slope coefficient = -0.109). Similarly, for participants eager to follow social norms, the proportion of sample who are vaccine non-compliant decreases at a greater rate (slope coefficient = -0.143) than those who are not eager to follow social norms (slope coefficient = -0.016) (Figure 4). On the other hand, Figure 3 demonstrates an increase in the proportion of sample who don’t know when they would get vaccinated (vs. delay) as local vaccination rates increased. For participants who are not eager to follow social norms, the proportion of sample never getting vaccinated increases at a greater rate (slope coefficient = 0.137) than those who are eager to follow social norms (slope coefficient = 0.107)

When accounting for social norms, although there are differences in the rate of decline in proportion of sample delaying or resisting vaccination in each figure, these differences are significant only when comparing the relationship for the sample resisting vaccination (vs. delaying) and for the sample who are not vaccine compliant (vs. delaying) (see Table 1).

**Figure 1: Relationship between vaccination delay vs. already vaccinated and local vaccination rates by social norms**

**Figure 2: Relationship between never getting vaccinated vs. delaying vaccination and local vaccination rates by social norms.**

**Figure 3: Relationship between not knowing when to get vaccinated vs. delaying vaccination and local vaccination rates by social norms.**

**Figure 4: Relationship between vaccination non-compliance (never getting vaccinated or don’t know when to get vaccinated) vs. delaying vaccination and local vaccination rates by social norms.**

**Table 1: Ordinary least square estimates predicting vaccination delay or non-compliance from vaccination rates and social norms**

| Variables | | (1) | (2) | (3) | (4) | (5) | (6) |
| --- | --- | --- | --- | --- | --- | --- | --- |
|  | | Delay vs. already vaccinated | Delay vs. as soon as possible | Delay vs. already vaccinated + as soon as possible | Never vs. delay | Don’t know vs. delay | Never + don’t know (non-compliance) vs. delay |
|  | | Coeff.  (Std. Err.) | Coeff.  (Std. Err.) | Coeff.  (Std. Err.) | Coeff.  (Std. Err.) | Coeff.  (Std. Err.) | Coeff.  (Std. Err.) |
| Local vaccination rates (%) | | -1.18**  (0.008) | -1.31**  (0.018) | -0.991**  (0.006) | -0.109**  (0.014) | 0.137**  (0.010) | -0.016  (0.012) |
|  | |  |  |  |  |  |  |
| *Social norms* | |  |  |  |  |  |  |
| You would be more eager to get vaccinated against COVID-19 if you see more people around you doing it (ref. = no) | | | | | | | |
|  | Yes | -1.40*  (0.633) | 0.625  (1.28) | 0.520  (0.445) | 2.56*  (1.21) | 0.910  (0.742) | 2.78*  (1.07) |
|  |  |  |  |  |  |  |  |
| Local vaccination rate $\times$ social norms | | 0.026  (0.018) | -0.002  (0.038) | -0.015  (0.013) | -0.134**  (0.038) | -0.030  (0.023) | -0.128**  (0.033) |
| Constant | | 76.9**  (0.271) | 85.7**  (0.616) | 58.2**  (0.197) | 40.1**  (0.455) | 17.7  (0.330) | 46.8**  (0.399) |
|  | |  |  |  |  |  |  |
| Observations | | 14,231 | 12,282 | 21,345 | 8,130 | 6,628 | 9,590 |
| F-statistic | | F(3,14227) = 9605.47 | F(3,12278) = 2284.91 | F(3,21341) = 13102.42 | F(3,8126) = 43.73 | F(2,6624) =  72.36 | F(3,9586) =  13.30 |
| Prob > F | | 0.000 | 0.000 | 0.000 | 0.000 | 0.000 | 0.000 |
| R^2^ | | 0.670 | 0.358 | 0.648 | 0.016 | 0.032 | 0.004 |

**p<0.01, *p<0.05
