# Supplementary material for: Timing of preventive behavior in the case of a new and evolving health risk: the case of COVID-19 vaccination
Source: Health Econ Rev. 2024 Feb 27;14:16. doi: 10.1186/s13561-024-00484-9 (PMC11344437; doi:10.1186/s13561-024-00484-9)
Supplement: Supplementary file 2 — Additional file 2. Online resource 2. Sensitivity analysis for COVID-19 vaccination timing categories. [file 13561_2024_484_MOESM2_ESM.docx]

**Online resource 2: Sensitivity analysis for COVID-19 vaccination timing categories**

**Table 1: Sensitivity analysis results for vaccination timing preference probit models**

|  |  | (1) | (2) |
| --- | --- | --- | --- |
| VARIABLES | | As soon as possible vs.  Already vaccinated | Later vs.  Sometime in 2021 |
|  |  | AME  (Std. Err.) | AME  (Std. Err.) |
| *Local vaccination rates (ref. = less than 30%)* | | | |
|  | 30 to 40% | 0.161** | -0.075 |
|  |  | (0.060) | (0.042) |
|  | 40% or more | -0.093 | -0.037 |
|  |  | (0.141) | (0.059) |
|  |  |  |  |
| *Trust in sources for reliable COVID-19 vaccines information* | | | |
| Among all the following sources, which ones would you trust more to give you reliable information on COVID-19 vaccines? | | | |
|  | European Union | -0.013 | -0.022 |
|  |  | (0.015) | (0.016) |
|  | National government | 0.036** | -0.069** |
|  |  | (0.013) | (0.023) |
|  | National health authorities | 0.020 | -0.107** |
|  |  | (0.014) | (0.019) |
|  | Local and regional public authorities | 0.051** | -0.044 |
|  |  | (0.014) | (0.025) |
|  | Health professionals, doctors, nurses, and pharmacists | -0.027** | -0.045** |
|  |  | (0.009) | (0.017) |
|  | Media (television, radio, newspapers) | 0.033* | -0.022 |
|  |  | (0.014) | (0.024) |
|  | Websites | 0.003 | 0.044 |
|  |  | (0.019) | (0.023) |
|  | Online social networks | 0.005 | 0.028 |
|  |  | (0.018) | (0.028) |
|  | People around you (colleagues, friends, and family) | 0.014 | 0.030* |
|  |  | (0.017) | (0.013) |
|  |  |  |  |
| *Social norm* | |  |  |
| You would be more eager to get vaccinated against COVID-19 if you see more people around you doing it (ref. = no) | | | |
|  | Yes | 0.044** | -0.119** |
|  |  | (0.014) | (0.023) |
|  |  |  |  |
| *Vaccine safety* | | | |
| To what extent do you agree or disagree with each of the following statements? | | | |
| COVID-19 vaccines are being developed, tested, and authorized too quickly to be safe. (ref. = totally disagree/tend to disagree) | | | |
|  | Totally agree/tend to agree | 0.0220* | 0.094** |
|  |  | (0.011) | (0.018) |
|  | Don’t know | -0.003 | -0.072 |
|  |  | (0.014) | (0.040) |
|  |  |  |  |
| COVID-19 vaccines could have long term side effects that we do not know yet. (ref. = totally disagree/tend to disagree) | | | |
|  | Totally agree/tend to agree | 0.027** | 0.031 |
|  |  | (0.008) | (0.021) |
|  | Don’t know | -0.012 | -0.147** |
|  |  | (0.011) | (0.026) |
|  |  |  |  |
| *Risk understanding* | | | |
| To what extent do you agree or disagree with each of the following statements? | | | |
| You can avoid being infected by COVID-19 without being vaccinated. (ref. = totally disagree/tend to disagree) | | | |
|  | Totally agree/tend to agree | 0.034** | 0.066** |
|  |  | (0.010) | (0.018) |
|  | Don’t know | 0.004 | 0.039 |
|  |  | (0.018) | (0.020) |
|  |  |  |  |
| *Demographics* | | | |
| Age (ref. = 15 – 24 years) | | | |
|  | 25-39 years | -0.111* | -0.006 |
|  |  | (0.021) | (0.023) |
|  | 40-49 years | -0.189* | -0.017 |
|  |  | (0.022) | (0.026) |
|  | 50-64 years | -0.321** | 0.007 |
|  |  | (0.026) | (0.026) |
|  | 65 years and above | -0.498** | 0.049 |
|  |  | (0.029) | (0.035) |
|  |  |  |  |
| Gender (ref. = female) | |  |  |
|  | Male | 0.042** | -0.024 |
|  |  | (0.009) | (0.016) |
|  | In another way | 0.054 | 0.023 |
|  |  | (0.077) | (0.099) |
|  | Prefer not to answer | 0.045 |  |
|  |  | (0.138) |  |
|  |  |  |  |
| Age when full time education was stopped (ref. = 20 years or older) | |  |  |
|  | Up to 15 years | 0.0012 | -0.070 |
|  |  | (0.030) | (0.041) |
|  | 16-19 years | -0.007 | 0.043** |
|  |  | (0.017) | (0.015) |
|  | Still in full time education | 0.044** | 0.004 |
|  |  | (0.017) | (0.023) |
|  | Never been in full time education | 0.026 | 0.060 |
|  |  | (0.028) | (0.038) |
|  | Don’t know | 0.024 | 0.077 |
|  |  | (0.025) | (0.042) |
|  | Refusal | -0.001 | 0.009 |
|  |  | (0.046) | (0.064) |
|  |  |  |  |
| Type of community (ref. = town/city) | |  |  |
|  | Rural | -0.023 | 0.022 |
|  |  | (0.012) | (0.014) |
|  |  |  |  |
| Children in house (ref. = no) | |  |  |
|  | Yes | 0.043** | -0.002 |
|  |  | (0.011) | (0.013) |
|  | Don’t know | 0.093** | -0.027 |
|  |  | (0.028) | (0.033) |
|  | Refusal | 0.016 | 0.025 |
|  |  | (0.021) | (0.030) |
|  |  |  |  |
| Observations | | 16,177 | 5,163 |
| Pseudo R^2^ | | 0.157 | 0.068 |
| Log pseudolikelihood | | -9357.28 | -3244.10 |

Estimates are average marginal effects (AME)

Standard errors in parentheses

** p<0.01, * p<0.05
