# Supplementary material for: Timing of preventive behavior in the case of a new and evolving health risk: the case of COVID-19 vaccination
Source: Health Econ Rev. 2024 Feb 27;14:16. doi: 10.1186/s13561-024-00484-9 (PMC11344437; doi:10.1186/s13561-024-00484-9)
Supplement: Supplementary file 3 — Additional file 3. Online resource 3. Correlation between vaccination attitudes in general and the timing of COVID-19 vaccinations. [file 13561_2024_484_MOESM3_ESM.docx]

**Online resource 3: Correlation between vaccination attitudes in general and the timing of COVID-19 vaccinations**

**Table 1: Pairwise correlations between vaccination attitudes in general and the timing of COVID-19 vaccinations**

|  | **Pairwise correlations** | | | | | |
| --- | --- | --- | --- | --- | --- | --- |
| **VARIABLES** | **Delay**  **vs.**  **already** | **Delay**  **vs.**  **as soon as possible** | **Delay**  **vs.**  **already + as soon as possible** | **Never**  **vs.**  **delay** | **Don’t know vs.**  **delay** | **Never + Don’t know**  **vs.**  **delay** |
| To what extent do you agree or disagree with the following statements about vaccines in general | | | | | | |
| Vaccines are safe | 0.064  (0.000) | 0.077  (0.000) | 0.064  (0.000) | 0.013  (0.236) | 0.141  (0.000) | 0.066  (0.000) |
| Vaccines are effective | 0.078  (0.000) | 0.093  (0.000) | 0.080  (0.000) | 0.074  (0.000) | 0.174  (0.000) | 0.114  (0.000) |

Significance levels in parenthesis
